# App-based symptoms screening with Xpert MTB/RIF Ultra assay used for active tuberculosis detection in migrants at point of arrivals in Italy: The E-DETECT TB intervention analysis

**DOI:** 10.1371/journal.pone.0218039

**Published:** 2019-07-01

**Authors:** Lucia Barcellini, Emanuele Borroni, Claudia Cimaglia, Enrico Girardi, Alberto Matteelli, Valentina Marchese, Giovanna Stancanelli, Ibrahim Abubakar, on behalf of the members of the E-Detect TB Consortium, Daniela Maria Cirillo

**Affiliations:** 1 Emerging Bacterial Pathogens Unit, Division of Immunology and Infectious Diseases IRCCS San Raffaele Scientific Institute, Milano, Italy; 2 Clinical Epidemiology Unit, National Institute for Infectious Disease “Lazzaro Spallanzani”–IRCCS, Rome, Italy; 3 Institute of Infectious and Tropical Diseases, Infectious Disease, University of Brescia, Brescia, Italy; 4 Tuberculosis Section, Health Protection Agency Colindale and University College London, London, United Kingdom; The University of Georgia, UNITED STATES

## Abstract

**Background:**

From 2014 to 2017, the number of migrants who came to Italy via the Mediterranean route has reached an unprecedented level. The majority of refugees and migrants were rescued in the Central Mediterranean and disembarked at ports in the Sicily region. Rapid on-spot active TB screening intervention at the point of arrival will cover most migrants arriving in EU and by detecting TB prevalent cases will limit further transmission of the disease.

**Material and methods:**

Between November 2016 and December 2017 newly arrived migrants at point of arrivals in Sicily, were screened for active Tuberculosis using a smartphone application, followed in symptomatic individuals by fast molecular test, Xpert MTB/RIF Ultra, on collected sputum samples.

**Results:**

In the study period 3787 migrants received a medical evaluation. Eight hundred and ninety-one (23.5%) reported at least one protocol-defined Tuberculosis symptom. Fifteen (2.7%) were positive to at least one microbiological test revealing a post-entry screening prevalence rate of 396 per 100.000 individuals screened (95% CI: 2.22–6.53). In logistic regression analysis, those with cough and at least one other symptom had an increased probability of testing positive compared to persons with symptoms other than cough. Whole-genome-sequencing demonstrate two separate cases of transmission.

**Discussion:**

To our knowledge this study reports first-time results of an active TB case finding strategy based on on-spot symptom screening using a smartphone application, followed by fast molecular test on collected sputum samples. Our preliminary findings reveal a post-entry screening prevalence rate of 396 per 100.000 individuals screened (95% CI: 2.22–6.53).

## Introduction

From 2014 to 2017, the number of migrants who came to Italy via the Mediterranean route has reached an unprecedented level, with a peak of more than 181,400 new arrivals in 2016. In the same period, 29,595 and 21,653 migrants entered by sea Greece (eastern Mediterranean route) and Spain (west Mediterranean route), making Italy the main point of entrance in Europe for refugees and migrants[1] [2]. The migrant population, departing mainly from Libya, includes newly arrived individuals, who subsequently either claim asylum or passing through Italy move to other EU countries[3].

Since the last two decades migration from high tuberculosis (TB) burden countries have contributed to slowing down the decreasing trend in TB incidence in European countries[4]. In low TB incidence EU/EEA, these trends are mainly affected by migration dynamics with TB incidence rates among foreign-born individuals usually several times higher than among non-foreign-born individuals[5]. Italy is a low TB incidence country, with a notification rate of 6.6 cases per 100,000 population and a prevalence of 4032 TB cases in 2016[5]. Since 2009, more than 50% of TB cases notified each year (and most of MDR TB cases) have occurred in the foreign-born population[6]. Hence, screening for TB among newly arrived migrants and asylum seekers represents a cornerstone of tuberculosis prevention and control strategies[7].

In 2015 to face the disproportionate migratory pressures, Italian National Authorities agreed to implement the so-called “hotspot-hubs” approach[8]. The “hotspot-hubs” approach aims to identify, register and properly process (including relocation and enforced return decisions for irregular migrants) new arrivals in few designated centers at key arrival points. In 2017 four “hotspots” (Lampedusa, Pozzallo and Trapani in Sicilian region and Taranto in Apulia) were operational, with a total official capacity of approximately 1,600 places. However, as the number of new arrivals has exceeded their capacity, mobile “hotspot” units were organized in Sicily and South Italy for identification and assistance purpose [9]. After completing the identification procedures in place, migrants applying for refugee status are usually transferred within two or three days to regional first-line reception centers (so-called “hubs”) where they wait for the decision on their application.

While the national guideline for screening refugees has recently been reviewed[10], the use of a national protocol on TB screening procedures is difficult to implement and highly-demanding. Nationwide data for the number of asylum seekers screened, and the active TB cases found in newly arrived refugees are scarce in Italy, mainly due to a fragmented reception and public health system. The national law requires notification of tuberculosis cases only. Different approaches are adopted by both the Italian regions and by the different health-care providers in the reception centers. In hotspots active case-finding is based on TB symptoms screening, while in first aid reception centres interventions are often limited to passive case finding, performed in symptomatic migrants who report themselves to health centres or who visit outpatient clinics for unrelated medical conditions. Microbiological or radiological investigations of suspected cases among new arrivals at the hotspot required authorised transport to local hospital, limiting the number of people successfully screened at arrival centres.

An Early DETECTion of tuberculosis consortium (E-DETECT TB) has recently been formed with the support of the Health program of the European Union bringing together national public-health agencies, academic centres and TB experts, with the aim to face the disproportionately high disease-burden of TB in vulnerable groups in European countries by using trans-national evidence-based intervention.

The aim of the study is to report the data on an innovative active TB case finding strategy to screen newly arrived migrants at point of arrivals in Sicily, based on symptom screening through a smartphone application, the E-detect App and on-spot sputum analysis relying on fast, sensitive and specific molecular assay such as the Xpert Ultra. (Cepheid, Sunnyvale, CA, USA)[11].

## Material and methods

### E-Detect TB App

One of the main challenges in screening activities in non-healthcare settings is the need for fast data recording and prompt sharing of clinical records with the referral hospital as well as with relocation sites.

Digital technologies are changing healthcare delivery globally. In TB field there is increasing recognition that digital technologies can support TB treatment adherence in diverse settings[12].

An electronic-health (eHealth) system called E-DETECT TB has been developed to address the need for a fast and user-friendly mobile tool to log screening results directly at the point of arrival and at relocation sites.

E-Detect TB App is a m-Health system[13] that helps health-care staff to perform active and latent TB screening practice according to the World Health Organization (WHO) recommendations [14] [15].

The system includes three components. A touch-screen icon-based application for Android smartphone, structured in five modules (Questionnaire, Smear, Diagnostics, Latent TB infection, Treatment and follow-up). To record information, the user can click on the relevant icons or take photos. The application additionally allows the user to verbally record and save any further comments if necessary. At the end of the questionnaire all data collected is automatically transmitted to a Web database. The Web database is a java-based software system with secure access to the medical staff of the reference hospital. The system receives all the information registered by the phone application during the visit and allows the clinical staff of the hospital to monitor the data collected. The data collected can be subsequently exported into an electronic database for scientific and epidemiological purposes. At the end of the e-questionnaire all data collected is automatically transmitted to the Medical Unit within the referral hospital. In the absence of cellular network coverage, it is still possible to use the application and store the data locally until an area with network coverage is reached.

### Microbiological procedures

#### OMNIgene-sputum

OMNIgene-sputum (DNA Genotek–Ontario)[16] is a highly stable non-toxic reagent that liquefies and decontaminates sputum samples at the point of collection, preserving MTB viability at ambient temperatures and minimizing contamination and cross-contamination. All samples were decontaminated onspot or within a day from the sputum collection using OMNIgene-sputum, according to the manufacture instruction[17]. Briefly, the specimens were mixed with an equal volume of OMNIgene-sputum and stored at ambient temperature. Samples in OMNIgene-sputum were shipped at ambient temperature to the Emerging Bacterial Pathogen Unit at san Raffaele Scientific Institute within a week. Upon arrival OMNIgene-sputum was removed by centrifugation at 2800 g for 20 min, and then the sediment was resuspended in 2 ml of phosphate buffer pH 6.7 for further processing.

#### Xpert MTB/RIF Ultra

TB screening approaches based on symptoms screening usually include a second step referral for further radiological/microbiological investigation. E-detect strategy uses fast molecular tests such as the Xpert MTB/RIF Ultra to confirm the diagnosis, thus resulting in a single step approach, feasible in outreach settings. Xpert MTB/RIF assay is an automated diagnostic test that can identify *Mycobacterium tuberculosis* DNA and resistance to rifampicin in less than 2 hours, compared to standard cultures that can take up to 6 weeks. Xpert Ultra incorporates two different multicopy amplification targets (IS6110 and IS1081) and uses improved assay chemistry and cartridge design, resulting in an approximately 1–log improvement in the lower limit of detection compared to the previous version of the test. The results of a first diagnostic accuracy study show that the sensitivity of Xpert Ultra is superior to that of the standard Xpert for tuberculosis case detection, especially in sputum smear-negative pulmonary tuberculosis[18], thus facilitating tuberculosis diagnosis at the earlier stages of disease. Sputum samples were tested by smear microscopy, solid and liquid culture, Xpert MTB/RIF (Xpert G4) and the new XpertMTB/RIF-Ultra (Xpert Ultra). Xpert G4 and Xpert Ultra assays were performed by adding decontaminant reagent to the collected sputum specimens, as per the manufacturers instruction[19]. Xpert G4 and Xpert Ultra present different grading system according to the number of cycles of amplification necessary to detect *M*. *tuberculosis* genome in the sample. Precisely Xpert Ultra has a new category “Trace” besides the four standard categories of “very low, low, medium and high” already present in the older Xpert G4. Smear microscopy was carried out using Ziehl-Neelsen (ZN staining) staining while the decontaminated sediment (0.5 ml) was inoculated in BBL MGIT tubes and incubated BACTEC MGIT960 instrument (BD Microbiology Systems, Sparks, MD, USA) and again 0.2 ml of decontaminated sediment was inoculated on Löwenstein-Jensen medium. Positive cultures were tested for ZN staining for the presence of Acid fast bacilli and subsequently tested by SD Bioline TB Ag MPT64 (Standard Diagnostics Inc.–South Korea) for identification of *M*. *tuberculosis complex* (Mtb) isolates. Positive Mtb cultures were tested for drug susceptibility testing for first line drugs.

#### Whole Genome Sequencing (WGS)

DNA was extracted for genomic analysis. Genomic DNA was extracted and purified by automated Maxwell system (Promega, Madison, WI, USA) Illumina (San Diego, CA, USA) technology was used for paired-end WGS applying the Nextera XT DNA sample preparation kit and the “benchtop” MiniSeq platform (300 cycles). Similarly, amplicons retrieved directly from amplification external chamber of the Xpert Ultra cartridges were sequenced for the detection of IS6110, IS1081 and rpoB gene to confirm the presence of Mtb DNA in samples categorized as “Trace”. The WGS transmission analysis was based on a multi locus sequence type (MLST) based on the analysis of allelic variants in the core-genome by use of Ridom SeqSphere+ (Ridom GmbH)[20]. Both this approach and formal contact investigation were carried out in order to confirm inter-human transmissions[20] [21]. We defined two isolates in cluster when the maximum number of allelic variants were less or equal to 5 units [20][21].

### E-Detect TB intervention

Between November 2016 and December 2017 individuals hosted in first line reception centres were screened for active TB, with the use of a e-questionnaire followed by sputum sample collection in symptomatic individuals. The screening was performed in 3 reception centers: the first aid reception centers of Mineo in Catania (whole period), the hotspot of Lampedusa and the temporary hotspot in Agrigento (from May 2017 to December 2017). The study population included asylum seekers defined as individuals seeking safety from persecution or serious harm in a country other than his or her own and awaits a decision on the application for refugee status[22].

All those arriving or already hosted in the centres were offered the opportunity to participate in a voluntary screening which was performed through a standardised E-questionnaire carried out by medical staff. The e-questionnaire assessed sociodemographic data including age, sex, country of origin, travel time as well as TB risk factors (including HIV status if known), previous TB disease and TB contact. Symptoms suggestive of TB were investigated: cough for more than 2 weeks duration, fever for more than 1-week duration, night sweats, weight loss and haemoptysis. On the spot sputum sample collection were requested if at least one symptom was present. Whenever possible collection of sputum sample were performed during the visit. On the spot decontamination was also performed using OMNIgene-sputum (DNA Genotek–Ontario)[16] on the collected samples[17]. Participants were asked to provide only one sputum specimen.

The aims and the methods of the study were explained to the participants verbally and through a printed leaflet in 4 different languages. Cultural mediators were presents in the centres when e-questionnaire were administered. Besides, regardless the participation to the study, information was provided on the main symptoms of TB disease and individuals were encouraged to visit the local health centres whenever these symptoms develop in the future. The screening intervention took place in agreement with local Authorities (Prefettura of Catania and Prefettura of Agrigento) and in collaboration with local health care providers and NGOs responsible for first support in the centres. The study was approved by the San Raffaele Institute Ethic Commission (protocol number 709624) and informed written consent was obtained from each subject before data collection. Microbiologically confirmed patients were address to the local Hospital for treatment and follow-up.

### Statistical analysis

Descriptive statistics are reported as proportions. For individuals reporting at least protocol-defined tuberculosis symptom, we investigated the association with lack of referral or refusal/non-attendance to sputum collection of relevant characteristics using multinomial logistic regression analysis through Relative Risk Ratios (RRR, sometimes interpreted as conditional odd ratios) and their 95% confidence intervals (95% CI; significance was set at p <0.05). Gender, age, TB incidence in the country of origin, presence of comorbidities, symptoms and time since arrival in Italy were included in the multivariable model which was also adjusted for center and screening period.

A univariate logistic regression was used to assess, among symptomatic individuals providing at least one sputum sample, variables associated with positivity of at least one microbiological test, calculating odds ratios (OR) and their 95% confidence intervals (95% CI).

Sensitivity and specificity for each of the molecular diagnostic tests were estimated as compared to culture (as a gold standard) and reported with 95% confidence intervals. Cohen’s kappa is used for assessing agreement between two molecular diagnostic tests (GeneXpert MTB/RIF vs GeneXpertMTB/RIF-Ultra).

Data management and statistical analysis was performed using IBM SPSS Statistics version 25 (IBM Corp., Armonk, N.Y., USA) and STATA version 13 (Stata Corp LP, College Station, TX, USA).

## Results

### Characteristics of the study population

Between November 2016 and December 2017, 3787 migrants, hosted at the participating reception centres, received a first medical evaluation, which included the TB symptom screening, at the participating reception centers. Characteristics of these individuals are summarized in Table 1. The median age was 22 years (IQR: 19–27), the vast majority were male, reflecting data on refugees arrivals to Italy which shows a prevalence of males (79%)[23]. Almost half of the study population arrived in Italy since less than one month (48.2%). Overall, 39 nationalities were represented; among these the most frequently found were: Nigeria (683, 18.4%), Eritrea (463, 12.2%), Ivory Coast (353, 9.3%), Senegal (316, 8.3%) and the Gambia (301, 8.0%). Nineteen individuals (0.5%) were HIV positive.

**Fig 1:**
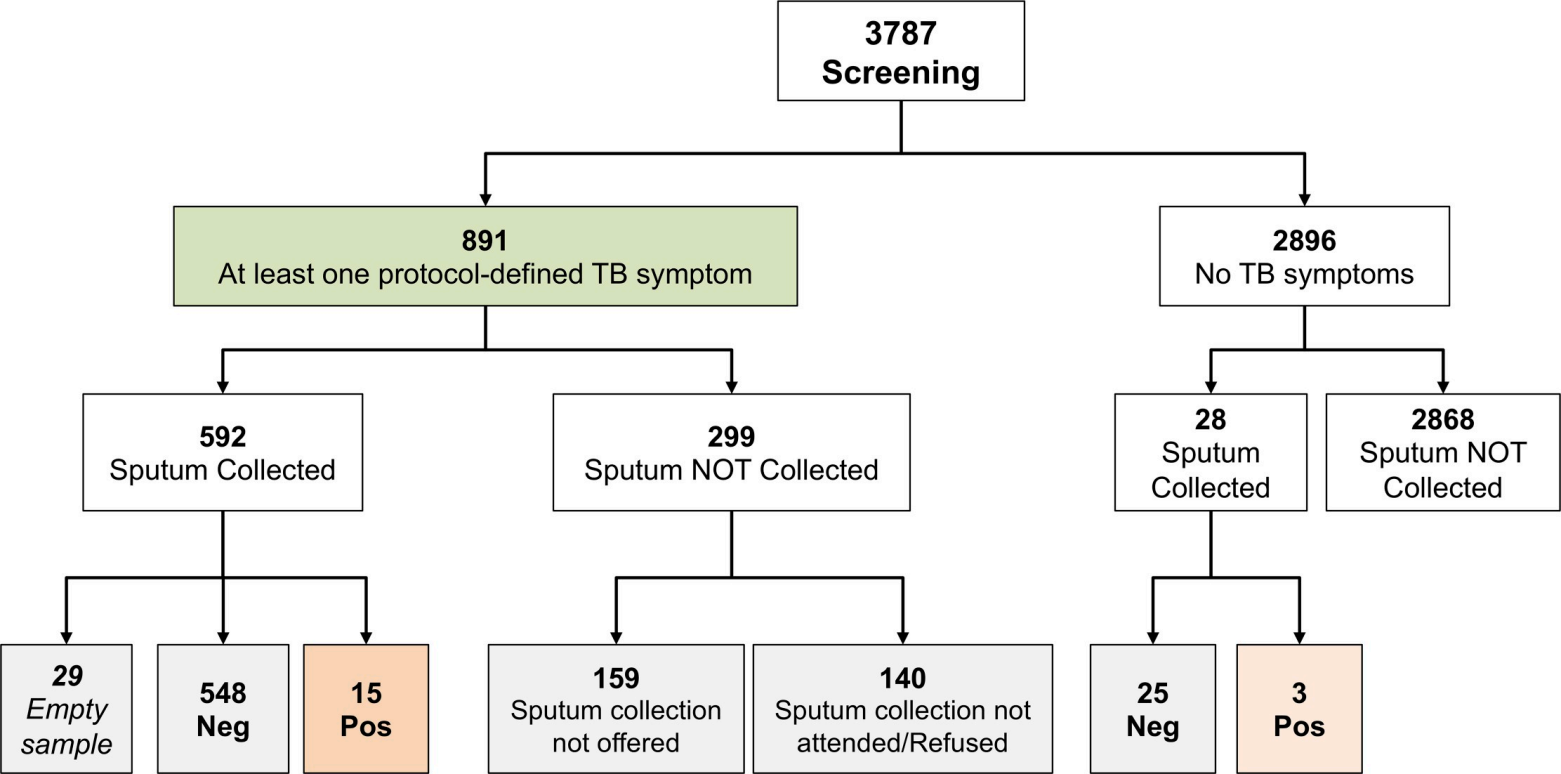
Study flow diagram.

The flow of enrolled individuals through the screening process is depicted in Fig 1. Eight hundred and ninety-one (23.5%) reported at least one protocol-defined tuberculosis symptom. Among them, 489 (54.9%) reported cough, 468 (52.5%) weight loss, 261 fever (29.3%), 218 (24.5%) night sweats, and 126 (14.1%) haemoptysis; 268 individuals (30.1%) reported 2 symptoms, and 176 (19.8%) 3 or more.

**Table 1:** Characteristics of 3787 individuals screened.

Characteristics	N.	%
Visit Location		
CARA Mineo (CT)	2666	70.4
Lampedusa (AG)	707	18.7
Siculiana (AG)	414	10.9
Gender		
Male	3263	86.2
Female	524	13.8
Age (years)		
≤20	1415	37.4
21–40	2267	59.9
>40	73	1.9
*Missing*	*32*	*0*.*8*
Age (years), median (IQR)	22 (19–27)	
Months since arrival in Italy		
<1	1824	48.2
1–4	818	21.6
5+	1045	27.6
*Missing*	*100*	*2*.*6*
Area of Origin*		
Northern Africa	416	11.0
Eastern Africa	539	14.2
Western Africa	2419	63.9
Central-Southern Africa	96	2.5
Western Asia	35	0.9
Southern Asia	275	7.3
*Missing*	*7*	*0*.*2*
TB incidence of Country of Origin^		
<100	1181	31.2
100–300	1817	48.0
>300	781	20.6
*Missing*	*8*	*0*.*2*
Previous TB	137	3.6
TB Contact	145	3.8
Predisposing conditions		
Smoker	1008	26.6
Alcohol	243	6.4
IDU	26	0.7
Diabetes	9	0.2
HIV	19	0.5

* Geographical regions used by the Statistics Division f the United Nations Secretariat (https://unstats.un.org/unsd/methodology/m49/)

^ Rates per 100 000 population. Global Tubercolosis Report—WHO 2016

IQR = Interquartile range; IDU = Intravenous Drugs Users

Among the 891 symptomatic individuals, 592 underwent sputum collection, 140 refused or not attended sputum collection. When an alternative diagnosis was already present a sample was not collected (159 individuals).

### Sputum yield and risk factors

Among the 563 persons with symptoms, for whom a valid sputum sample was available, 15 (2.7%) were positive for at least one microbiologic test. This yield represents 0.40% of migrants included in the study; a point prevalence of 400 per 100,000. In logistic regression analysis compared to persons with symptoms other than cough, those with cough and at least one other symptom had an increased probability of testing positive (OR = 4.41, p<0.10; Table 2) but this estimate is imprecise (95% CI: 0.97–20.11) and the association is not statistically significant at the conventional p-value<0.05[24].

**Table 2:** Factors associated with positive microbiologic test at univariable analysis in symptomatic individuals.

	N. TB pos/Tot	OR (95% CI)	p-value
Gender			
M	13/491	1	
F	2/72	1.05 (0.23–4.75)	NS
Age (years)*			
< = 20	7/199	1	
>20	8/361	0.62 (0.22–1.74)	NS
TB incidence of Country of Origin^			
<100	4/143	1	
100–300	9/277	1.17 (0.35–3.86)	NS
>300	2/143	0.49 (0.09–2.74)	NS
Symptoms			
Cough	2/116	1.69 (0.24–12.18)	NS
Cough + Others symptoms	11/252	4.41 (0.97–20.11)	0.056
Others symptoms	2/195	1	
Comorbidity			
0	9/376	1	
1+	6/187	1.35 (0.47–3.86)	NS
Previous TB*			
No	13/491	1	
Yes	1/22	1.75 (0.22–14.02)	NS
Months from arrival*			
<1	7/307	1	
1–4	1/108	0.40 (0.05–3.29)	NS
5+	7/138	2.29 (0.79–6.66)	NS

NS = Not Significant

* The sum could not add up to the total because of missing values

^ Rates per 100 000 population. Global Tuberculosis Report—WHO 2016.

### Yield among those with no symptoms

Among the 2896 individuals not reporting protocol defined TB symptoms, 28 non-symptomatic persons (0,9%) underwent sputum collection based on personal will and physician judgement: 5 of them reported previous active disease and 4 of them were recent contact of active TB patients and want to be tested. Out of 28 individuals, 3 had at least one microbiologic test positive. One of them was a HIV positive woman in antiretroviral therapy, who wanted to be tested as her daughter was currently affected by pulmonary TB. The second patient had active TB disease in 2016 but the length of the treatment was unknown. No information is available on the third positive individual.

### Head to head comparison between Xpert and Xpert Ultra

Among the 591 migrants with a sample collected, with or without symptoms, 18 were positive for at least one microbiologic test: 11 individuals had culture-positive sputum (two of them, were positive only to culture), 9 had Xpert G4 positive results and 16 samples had Xpert Ultra positive results. None was rifampicin-resistant based on phenotypic drug susceptibility testing and Xpert results (both Xpert G4 and Xpert Ultra)

Results of the comparison between Xpert and Xpert Ultra sensitivity and specificity are shown in Table 3. Sensitivities of Xpert Ultra and Xpert G4 were 81.8% and 54.6%, respectively, having culture as reference comparator. Specificities of Xpert Ultra and Xpert G4 for case detection were 98.7% and 99.5%. The overall agreement between the two tests was 0.71 (0.64–0.79 95% CI).

**Table 3:** Diagnostic performance of GeneXpert MTB/RIF and GeneXpert MTB/RIF-Ultra as compared to MGIT culture.

	Sensitivity (95% CI)	Specificity (95% CI)
**GeneXpert MTB/RIF-Ultra**	81.82% (52.30–94.86)	98.73% (97.40–99.38)
**GeneXpert MTB/RIF**	54.55% (28.01–78.73)	99.46% (98.44–99.82)

The two tests gave concordant results for 9 subjects, while discordant results were found in 7 subjects. All discordant results scored negative to the Xpert G4 and positive to the Xpert Ultra assay. Most of the discordant results showed positivity scoring Trace (6 Trace, 1 Very Low). The characteristics of subjects with discordant results are shown in Table 4.

**Table 4:** Xpert MTB/RIF G4 vs Ultra discordant results.

ID Patient	CULTURE	G4 (grade)	ULTRA (grade)	Patient characteristics	MTB DNA sequencing
1	Neg (MGIT+LJ)	Neg	**Pos (T)**	Night sweats	missing
2	**Pos (MGIT)**	Neg	**Pos (T)**	Cough(3 mo) + haemoptysis	IS6110, IS1081
3	**Pos (MGIT+LJ)**	Neg	**Pos (VL)**	Pregnant, cough/fever/ weight loss	IS6110, IS1081
4	Neg (MGIT+LJ)	Neg	**Pos (T)**	Cough /fever	IS6110
5	Neg (MGIT)	Neg	**Pos (T)**	weight loss	missing
6	**Pos (MGIT)**	Neg	**Pos (T)**	HIV /TB contact/asymptomatic	IS6110, IS1081
7	Neg (MGIT+LJ)	Neg	**Pos (T)**	Not confirmed	IS6110
	**3**	**7**	**7**		

Sequencing of amplicons was performed on specimens for which Xpert Ultra gave “Trace and Rifampicin indeterminate” results, Sequencing of the amplicons obtained from the 6 samples with apparent false-positive results showed the presence of insertion sequences (IS6110 and IS1081) and rpoB gene in all tested samples (for 1 sample the amplicon was not available) (Table 4)

Whole Genome Sequencing (WGS) analysis has been performed for all the culture positive samples. Genomes comparison demonstrated two separate cases of transmission probably occurred at the first aid reception centre of Catania.

## Discussion

The migrant population accounts for an increasing large proportion of TB cases in EU/EEA and challenges TB controls strategies. Currently two main TB control strategies has been applied among migrants: active TB cases finding using symptom screening followed by referral of symptomatic patients to hospital for further investigations, and CXR-based screening. However, both strategies are resource-demanding and difficult to implement at point of arrival due to logistic as well as security issues.

In the last few years, the Central Mediterranean route from North Africa to Italy has experienced an unprecedented increase in population movement[2]. The majority of refugees and migrants were rescued in the Central Mediterranean and disembarked at ports in the Sicily region. Rapid on-spot active TB screening intervention at the point of arrival will cover most migrant arriving in EU and by detecting TB prevalent cases will limit further transmission of the disease.

The present study report data of post-entry active TB screening of asylum seekers performed in Italian hotspots and primary reception centers located in Sicily using a smartphone application for data collection, followed by on-spot collection of sputum sample and and molecular rapid test using Xpert Ultra assay.

To our knowledge the study first report the active TB prevalence among newly arrived migrant in Sicily. Out of 3787 migrants receiving first medical evaluation, 891 reported at least one symptom of TB and were referred to sputum collection that was actually done for approximately 60% of them. Adherence to tuberculosis case finding interventions based on symptom screening in migrants and refugees/asylum seekers has been evaluated in previous studies conducted in primary care centers and mobile clinics in Italy. In these studies, diagnostic evaluation provided in TB clinics was completed by 30% of symptomatic individuals [25]. This suggest that on site provision of diagnostic test increase the adherence to screening procedure.

Our results reveal a post-entry screening prevalence rate of 3.96 per 1000 individuals screened (95% CI: 2.22–6.53). Several systematic reviews assessed the yield of active TB case among migrants. Screening yield was highly heterogenous across the studies reflecting the heterogeneity of post-arrival programs (type of migrant screened, timing and setting of the screening intervention, TB incidence in the country of origin and TB screening procedures)[26]. The overall yield of CXR to detect active TB in post-arrival settings was 350 cases/100.000[26]. Vanino et al. published data of CXR-based TB screening intervention among asylum seekers arriving in the “hub” of Bologna[27]. The study showed a post-entry screening prevalence rate of 535 (95% confidence interval, 317–844) per 100000 individuals screened[27]. Thus, the yield of our intervention appears to be of the same order of magnitude of other interventions using different approaches.

CXR screening has been reported to have higher sensitivity of (98%)compared to symptoms screening (78%)[28]. On the other hand, CXR has a poor specificity for TB, and radiographic findings need to be confirmed by laboratory tools[28]. Our screening approach, by using symptoms screening, may miss some prevalent asymptomatic TB cases. However, it will reduce the use of local resource and therefore is more suitable to be applied in emergency settings, where radiographic facilities are not available, and transport is limited.

The 23.5% of the population screened reported at least one symptom, cough was the most frequently reported (54.9%). 15 cases of TB were microbiologically detected. In the logistics regression analysis individuals who reported cough associated to other symptoms seem to have a greater probability of TB compared to migrants reporting one of the symptoms other than cough (even if the estimate is imprecise and the association does not reach statistical significance). This finding is consistent with the fact that our approach allows diagnosis of highly symptomatic patients while may be less effective for sub-clinical and pauci-symptomatic TB forms.

Two TB cases were found among asymptomatic persons demonstrating the limits of symptom-based screening. One of them was a HIV positive woman in antiretroviral therapy, who wanted to be tested as active pulmonary TB was confirmed in the daughter. The second had active TB disease in 2016. He was previously hospitalized and treated but duration of anti-tuberculosis treatment was uncertain. A third patient reported an Xpert Ultra positive (trace), Xpert G4 and culture negative sputum. He was a young asymptomatic male, who did report neither past disease nor previous TB contact but willing to be screened. As per WHO recommendations [29] in absence of symptoms as well as predisposing factors to TB development, Ultra were repeated on a second and third sputum specimen. Trace positivity was not confirmed.

The main difference between the strategy here described and the majority of TB screening approaches based on symptoms screening is the use of fast molecular tests, as the Xpert MTB/RIF Ultra (Xpert Ultra), to confirm the diagnosis. Xpert MTB/RIF assay (Xpert G4 and Xpert Ultra) is an automated diagnostic test that can identify *Mycobacterium tuberculosis* DNA and resistance to rifampicin in less than 2 hours. Results of first diagnostic accuracy study show that the sensitivity of Xpert Ultra is superior to that of the Xpert G4 for tuberculosis case detection especially in sputum smear-negative pulmonary tuberculosis[18]. In clinical practice, the high sensitivity of Xpert Ultra could facilitate diagnosis of tuberculosis at earlier stages of disease. In our study Xpert Ultra sensitivity was higher than that of the standard Xpert for TB case detection in participants with culture positive sputa (81.8% vs 54.6%, respectively). Specificities of Xpert Ultra and Xpert for case detection were 98.7% and 99.5% respectively. The two tests show an overall good agreement. Discordant results were found in 7 subjects: they all scored negative to the Xpert G4 and positive to the Xpert Ultra assay. In most of the discordant samples, the semiquantitative Xpert Ultra results correspond to the lowest bacillary burden (trace). Three of them were culture positive-Xpert Ultra positive samples, which will be missed out by Xpert G4 (one of them was a HIV patient). Four samples were culture negative-Xpert G4 negative but Xpert Ultra positive samples. One of them was an asymptomatic individual, whose positivity were not confirmed by further microbiological analysis. None of them reported past TB disease. False-negative cultures from over-decontamination has been also propose as possible explanations for a positive nucleic-acid amplification test result and negative sputum cultures. In this regard the use of OMNI-gene sputum to perform on spot decontamination may have caused an over-decontamination as indicated by the low rate of contaminated culture found (1.8% in liquid medium)

WGS analysis has been performed for all the culture positive samples. The sequencing results demonstrate two separate case of potential transmission most probably occurred in the Catania Hubs. The first transmission identified (1 allelic variant), confirmed by WGS and epidemiological links, was between two relatives (a mother and the daughter) sharing the same room. In the second case (no allelic variant found) the two patients involved were two young men from Guinea and Liberia, they are not home-contact and apparently, they do not know each other. At the time of the diagnosis they have been hosted in the centre for 6 and 10 months respectively. No transmission were found in the hotspot of Lampedusa or Agrigento or between people hosted in different reception centers. Notably, the reception Centre of Catania, is the only hub included in the study. While accommodation in hotspot is strictly limited to the time need to carry out operations to defined the legal position, hubs are structures where migrants formalised their asylum request and wait for the approval. Social mixing, poor housing condition and overcrowding could facilitate the transmission of TB, therefore active TB case finding and systematic contact investigation should be implemented.

The study has several limitations. The foremost limitation is that the study design did not allow to measure to what extent our approach may have increase the detection rate of active TB and/or reduced diagnostic delay. We could not find data on the number of TB cases notified before or after our intervention during a similar period of time at the enrolment centers. The small number of active TB cases identified limits our ability to investigate risk factors for a positive test. Similarly, we are unable to formally compare this strategy with CXR screening as this was not performed.

To our knowledge this study reports for the first-time results of an innovative active TB case finding strategy based on-spot symptoms screening using a smartphone application followed by fast molecular test on sputum samples collected. The use of a phone application standardised data collection, allowed screening in the “field” and transfer of patients information directly to the referral Hospital. Fast molecular assays facilitated rapid identification of TB cases and prompt referral to the hospital in case of TB detection. It has been reported that TB screening at point of arrival is more acceptable in migrant patients. Moreover, single step for screening test, outreach settings and quick turnover of results increase the effectiveness of screening approach among migrants[25] Our screening approach offering TB symptoms screening and immediate on spot microbiological analysis may reduce the loss in the cascade of care. In our setting Xpert Ultra is more sensitive compared with the standard version of the test. Major advantages of the Xpert MTB/RIF assay in this setting are the rapid results, minimal technical training required to run the test, and the future possibility to perform the cartridge base molecular test directly on spot by using platform such as the portable Xpert OMNI.

## Supporting information

S1 Fig Survey questionnaires used in the study. The questionnaire includes individuals' personal data, date of arrival in the centre, past medical history, past TB history, risk factors for TB and protocol-defined symptoms of TB (cough, fever, haemoptysis, night sweats and weight loss) Questionnaire is available in three languages (English, French, and Arabic).
(DOCX)
