# Supplementary figures and images for: App-based symptoms screening with Xpert MTB/RIF Ultra assay used for active tuberculosis detection in migrants at point of arrivals in Italy: The E-DETECT TB intervention analysis
Source: PLoS One. 2019 Jul 1;14(7):e0218039. doi: 10.1371/journal.pone.0218039 (PMC6602175; doi:10.1371/journal.pone.0218039)

**
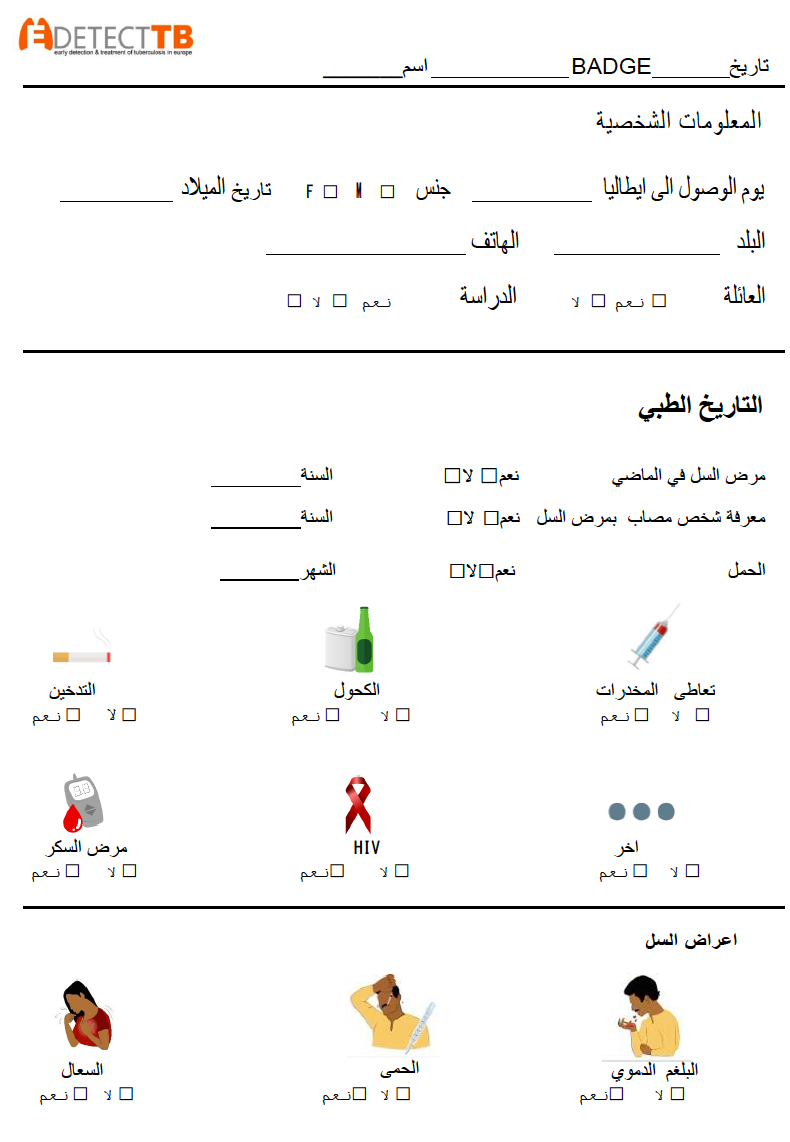
**

Supplement: S1 Fig — The questionnaire includes individuals' personal data, date of arrival in the centre, past medical history, past TB history, risk factors for TB and protocol-defined symptoms of TB (cough, fever, haemoptysis, night sweats and weight loss) Questionnaire is available in three languages (English, French, and Arabic). (DOCX) [file pone.0218039.s001.docx]
